# *Salmonella* and Eggs: From Production to Plate

**DOI:** 10.3390/ijerph120302543

**Published:** 2015-02-26

**Authors:** Harriet Whiley, Kirstin Ross

**Affiliations:** Health and the Environment, Flinders University, GPO Box 2100, Adelaide, 5001, Australia; E-Mail: Kirstin.Ross@flinders.edu.au

**Keywords:** *Salmonella*, salmonellosis, public health, risk assessment, caged, free range, organic, food handling, foodborne illness

## Abstract

*Salmonella* contamination of eggs and egg shells has been identified as a public health concern worldwide. A recent shift in consumer preferences has impacted on the egg industry, with a push for cage-free egg production methods. There has also been an increased desire from consumers for raw and unprocessed foods, potentially increasing the risk of salmonellosis. In response to these changes, this review explores the current literature regarding *Salmonella* contamination of eggs during the production processing through to food handling protocols. The contamination of eggs with *Salmonella* during the production process is a complex issue, influenced by many variables including flock size, flock age, stress, feed, vaccination, and cleaning routines. Currently there is no consensus regarding the impact of caged, barn and free range egg production has on *Salmonella* contamination of eggs. The literature regarding the management and control strategies post-collection, during storage, transport and food handling is also reviewed. Pasteurisation and irradiation were identified as the only certain methods for controlling *Salmonella* and are essential for the protection of high risk groups, whereas control of temperature and pH were identified as potential control methods to minimise the risk for foods containing raw eggs; however, further research is required to provide more detailed control protocols and education programs to reduce the risk of salmonellosis from egg consumption.

## 1. Introduction

Worldwide, *Salmonella* is one of the most prevalent causes of foodborne illness [1,2]. Globally, the annual incidence of foodborne salmonellosis is conservatively estimated at 80.3 million cases [3], but other estimates range from 200 million to 1.3 billion cases [4]. In the United States alone it was estimated that non-typhoidal *Salmonella* spp. are responsible for 1 million cases of domestically acquired foodborne illness annually [5]. A study from the European Union estimated that only 1 out of every 57 cases of salmonellosis is reported. This study also demonstrated that the annual incidence of salmonellosis in each of the European Union member states varied between 16 and 11,800 per 100,000 people and that the incidence of salmonellosis in each country correlated significantly with the presence of *Salmonella enterica* serotype Enteritidis in laying hens, suggesting this was the primary source of infection [6].

Contamination of eggs and eggshells has been identified as one of the major causes of foodborne *Salmonella* [1]. In the United States between 1985 and 2002 contamination of eggs was identified as the source of 53% of all cases of *Salmonella* reported to the Centre for Disease Control and Prevention (CDC) [7]. The two most commonly identified causative agents of foodborne salmonellosis are *S. enterica* serotypes Typhimurium and Enteritidis [2]. Both serotypes have the ability to colonise the reproductive organs of hens (the oviduct and ovary) and are major causes of foodborne illness. Globally *Salmonella* Enteritidis is more commonly linked to contaminated eggs, except in Australia, where the majority of egg-related foodborne salmonellosis is caused by *Salmonella* Typhimurium [8,9,10].

*Salmonella* contamination of eggs is a complex issue that is influenced by many variables, making it difficult to implement appropriate management strategies. There are two pathways for eggs to become internally contaminated with *Salmonella*. Direct contamination occurs during the formation of an egg in the reproductive tract of hens (including the ovary and oviduct); whereas, indirect contamination occurs after an egg has been laid and *Salmonella* contaminating the outside of the egg penetrates through the shell membrane [1,11]. These pathways for contamination can be influenced by the egg production process, storage, handling and food preparation [7,12,13,14].

In recent years, there has been shift driven by consumers for more humane methods of egg production, causing a shift from conventional battery cage housing systems to free-range production [15]. There has also been a shift in consumer eating habits with increasing demand for raw and unprocessed foods [16,17]. The increasing popularity of unprocessed home-made foods containing raw eggs such as mayonnaise, certain sauces and raw egg based deserts like ice-cream, tiramisu and even milkshakes potentially increases the risk of salmonellosis [17,18,19,20].

Currently, publications assessing the impact that various methods of egg production have on *Salmonella* contamination are conflicting, which makes it difficult to implement informed legislation to ensure food safety [21]. This manuscript reviews the current knowledge regarding *Salmonella* contamination of caged, barn and free range egg production processes. It also explores the various methods for control during production and at the point of use and how this can be influenced by the consumer. Discussion of current management policies and identification of gaps in knowledge will help inform future management protocols to ensure the safety of consumers.

## 2. Egg Production Processes

Studies comparing *Salmonella* contamination in the different egg production processes have yielded conflicting and inconsistent evidence. This is due to the complexity of confounding factors and variables. These factors include flock size, flock age and stress caused by rehousing, weather, transport, initiation of egg lay and moulting [22]. Another difficulty with interpreting results is the variation in the contamination pathways. Factors affecting *Salmonella* contamination of eggs differ for direct contamination within the ovaries and indirect contamination of environmental samples [23].

Currently, the conflicting evidence surrounding the influence of housing systems on *Salmonella* contamination still causes serious debate. A study by the European Food Safety Authority tested faecal and dust samples from 5000 egg production sites across 25 European countries and concluded that cage flock holdings were more likely to be contaminated with *Salmonella* [24]. However, a more recent review by Holt *et al.* [22] concluded there was no general consensus as to which egg production housing system resulted in less *Salmonella* contamination. This review was criticized by Greger [25] who stated that Holt *et al.* [22] had misrepresented data published by The European Food Safety Authority by only citing individual studies from four countries, representing less than a quarter of the total study. Greger’s rebuttal stated that presenting individual studies rather than a cumulative review of results allowed readers to have a more in depth and informative comparison of results. By presenting the data this way it raised the question as to why five of the studies showed higher incidence of *Salmonella* in free range housing compared to caged housing, which was contradictory to the rest of the studies. Answering this question may provide a better insight into the factors which may be promoting *Salmonella* contamination. These publications demonstrate the complexity of this issue and indicate that there is not a singular answer. As the egg production processes are currently undergoing rapid change it is important to identify the specific factors that promote *Salmonella* contamination as this will ensure the best management practices for the future.

## 3. Direct Contamination

Internal contamination of eggs with *Salmonella* occurs in the reproductive organs during egg formation [26]. Both *S.* Enteritidis and *S*. Typhimurium have been demonstrated to have the ability to colonise the reproductive tract of hens [8], however *S*. Enteritidis is more frequently isolated from the internal contents of eggs due to its ability to adhere better to reproductive tract mucosa compared to *S.* Typhimurium [9]. Internal contamination is an important issue, not only for human health, but for the egg production industry, as Gantois *et al.* [8] observed that hens infected with *Salmonella* had decreased egg production which did not improve within 2 weeks post-infection. Currently there are limited studies investigating the effect of different housing systems on this contamination pathway. Gast *et al.* [13] compared *Salmonella* contamination of hens in conventional cages and colony cages enriched with perching, nesting and scratching areas. Hens were orally dosed with 1.0 × 10^7^ CFU of *S.* Enteritidis for 5 to 6 days prior to euthanisation and testing of internal organs. *S.* Enteritidis was detected at significantly higher frequencies in the livers, spleens, ovaries and oviducts of the hens housed in the conventional cages compared to the hens housed in the enriched cages. It was suggested this could be due to housing parameters such as stocking density or behavioural attributes might affect the susceptibility of hens to disseminated infection. However, another study by Gast *et al.* [23] demonstrated experimentally that there was no significant difference in the rate of transmission of *S.* Enteritidis from infected hens to healthy hens housed in with conventional cages or enriched cages. The effect of housing on the transmission of *S.* Enteritidis infection was also explored by De Vylder *et al.* [27]. Four housing systems were explored using experimentally infected hens. This included a conventional battery cage, a furnished cage (most similar to an enriched cage), an aviary, and a floor system. The spread of infection between hens was slightly more in the aviary and floor housing systems compared to the two caged housing systems. This was partly reflected with the egg contamination as significantly more contaminated eggs were found in the aviary housing systems compared to two cage and floor housing systems. It was suggested that the increased spread of infection could be to inherent differences between the housing systems, including hygienic status, air quality and increased physical contact between birds.

## 4. Environmental Contamination

Numerous studies suggest that environmental sources present in free range housing have a lower incidence of *Salmonella* contamination compared to caged housing [12,24,28]. A Belgian study found that 30% (45/148) of dust samples and 30% (45/148) of faecal samples collected from caged housing were positive for *Salmonella;* whereas, only one out of 148 of dust samples and two out of 148 faecal samples collected from barn and free range housing were positive for *Salmonella* [12]. These results were supported by a UK study by Wales *et al.* [28] who found the incidence of *Salmonella* in environmental samples to be higher in caged housing (19%) compared to free range housing (10%). As noted already, the study by Recio *et al.* [24], which investigated the presence of *S.* Enteritidis in faeces and dust samples from 5310 egg production holdings across the European Union found that free range housing systems had significantly lower *Salmonella* contamination compared to caged housing systems. However conflicting evidence was presented by Parisi *et al.* [15] who used eighty-four certified *Salmonella*-free Bovan Brown hens to experimentally demonstrate that free range eggs had a higher incidence of *Salmonella* contamination compared to conventional battery cages. In this study 5/212 (2%) eggs sampled from three free range housings and 0/212 from three conventional battery cages tested positive for *Salmonella*. It was suggested that the higher *Salmonella* incidence in the free range housing was due to prolonged interaction between the hen and the egg after it has been laid, compared to cage systems when the egg is removed more quickly from the physical proximity to the hen.

## 5. Penetration of Eggs Post-Laying

Post-laying internal *Salmonella* contamination of eggs from environmental sources ocurrs through penetration of the shell membrane [11]. Miyamoto *et al.* [29] explored the potential of *Salmonella* to penetrate egg shells by immersing the eggs in *S*. Enteritidis and *S.* Typhimurium solutions at varying times post-laying. The highest incidence of internal *Salmonella* contamination occurred when eggs were between 15 min and 3 h post-laying (the shortest time period reported) and stored at 25 °C (compared with 3.25 h to 6 h, 1 day and 7 days post-laying). Refrigerating eggs at 4 °C for 15 min prior to *Salmonella* exposure significantly decreased penetration of the eggshell. It was suggested that this was due to reduced growth at the lower temperature. This indicates that refrigeration of eggs at collection may be a useful tool for minimising internal *Salmonella* contamination; however, realistically this is difficult to implement as it will not prevent any contamination that occurs in the housing prior to collection. The ability of *S*. Enteritidis and *S*. Typhimurium to penetrate eggshells was not significantly different. De Reu *et al.* [11] demonstrated experimentally that the age of the hen and eggshell characteristics such as area, shell thickness and number of pores does not significantly influence the eggshell penetration by *S*. Enteritidis. Another study by Messens *et al.* [30] used commercially available eggs and experimentally inoculated them with 2.71 log CFU of *S.* Enteritidis at 20 °C for 14 days. The rate of internal contamination was 6% for free-range, 16% of the conventional battery caged and 30% to 34% for the brown, organic, and omega-3-enriched eggs. Another trend observed in this study was that hens fed corncob mix had a higher incidence of penetrated eggshells compared with the hens given the standard feed, suggesting that feed type might affect eggshell permeability.

## 6. Production Control Measures

There are numerous methods that have been explored to control *Salmonella* contamination through the production process, one of the basic methods being routine cleaning and disinfection between flocks [28]. However, the effectiveness of these cleaning routines can be highly variable [14]. Wales *et al.* [28] investigated 12 *Salmonella*-contaminated caged layer houses post cleaning and disinfection and found that none of the 12 housings were completely *Salmonella*-free. Another study by Davies and Breslin [14] compared the effectiveness of cleaning and disinfection in free range, barn and cage layer housing and found that there was a decrease in *Salmonella* contamination observed in free range housing although the soil remained contaminated, but in the barn and cage housing significant contamination remained on the surfaces of buildings and equipment. Anecdotally it has also been suggested that there may be reduction in contamination as a result of modern farming methods. For example modern barn systems disposing of faecal material via manure belts would have lower contamination compared with older barn systems which would allow faecal material to pool until restocking.

The cross contamination of wildlife vectors has also been identified as a mechanisms for recontamination of housing [14,28,31,32]. For example, *S.* Enteritidis was found to be more commonly isolated from caged housing compared to *S.* Typhimurium. One suggestion for the reasoning behind this was the strong correlation between *S.* Enteritidis and rodent activity which was not observed for *S.* Typhimurium. Other animals which have been identified as carriers for *Salmonella* causing recontamination of farms include mice, rats, foxes, cats, flies, litter beetles, ground beetles and centipedes [31,32]. Differences in egg production housings systems, climate and region also influence the effectiveness of biosecurity measures implemented against each of these animal vectors, affecting the success of remediation and prevention measures [22].

Vaccination of hens has had varying success against *Salmonella* infection, depending on the vaccine and the *Salmonella* serotype. Berghaus *et al.* [33] demonstrated that a vaccine containing killed *S.* Typhimurium, *S.* Enteritidis, and *S. enterica* serotype Kentucky increased the immunity of the hens and their progeny against these particular serotypes; however, it did not decrease the incidence of *Salmonella* in environmental samples taken from the housing. Another study by Arnold *et al.* [34] found that vaccination did not influence the proportion of hens shedding *S.* Enteritidis and *S.* Typhimurium; however, it did significantly decrease the incidence of *S.* Enteritidis and *S.* Typhimurium present on eggshells compared to the non-vaccinated hens.

Antibiotic treatment is a controversial method for the control of *Salmonella* due to the early emergence of antibiotic resistant populations [35]. A study by Li *et al.* [36] tested fecal samples collected from a commercial layer house over a 78 week period. Using PFGE they characterized 45 different *Salmonella* isolates with known serovars. Of these 45 isolates, 16 (35%) were resistant to at least one of the 15 antibiotics tested against. This included resistance against tetracycline, ampicillin, streptomycin, and ceftiofur which are widely used in the treatment of human systemic salmonellosis. A more recent study investigated the presence of antibiotic resistant genotypes of *Salmonella* isolated from broiler hens found that more than 43% of the isolates were resistant to ampicillin, amoxicillin-clavulanic acid, ceftiofur, cefoxitim, and ceftriaxone [37]. This was supported by Adesiyun *et al.* [38] who isolated 84 isolates of *Salmonella* from egg productions processes in Trinidad and Tobago, Grenada, and St. Lucia and found that all of the 84 isolates displayed resistance to one or more of the seven antimicrobial agents tested. A high frequency of resistance was observed against erythromycin, streptomycin, gentamycin, kanamycin, ampicillin, and tetracycline. The presence of antibiotic resistant strains is highly relevant to food safety and public health with regard to treatment of the more invasive cases of salmonellosis [37].

Another control method was explored by Fiorentin *et al.* [39] who demonstrated that orally treating hens infected with *S.* Enteritidis with bacteriophages isolated from free-range hens was found to significantly reduce the contamination of *S.* Enteritidis found in the caeca. A 3.5 order of magnitude reduction of *S.* Enteritidis CFU/g of caecal matter was observed five days after treatment with the bacteriophage and samples collected up to 25 days after treatment continue to have contamination concentrations compared to infected hens not treated with the bacteriophage.

## 7. Post-Collection Control Methods

The benefits of current egg washing technology has been debated due to concerns the process may transfer *Salmonella* from the egg shell surface into the contents of the egg [40]. There is also the concern that washing can spread *Salmonella* causing cross contamination [41]. Hutchison *et al.* [42] demonstrated experimentally that washing contaminated eggs under optimum conditions (conveyor belt speed of 111 cm/min, prewash water was 44 °C and 138 kPa, wash water was 44 °C, 262 kPa and contained 3 g/L of chlorowash (a chlorine based disinfectant, rinse water was 48 °C, 262 kPa and contained 2.5 ml/L of Quat 800 rinse water agent and post-washing air eggs drying for 2 min at 42 °C) resulted in a 5 log reduction of *Salmonella* CFU on the shell surface and *Salmonella* was not detected from the internal contents of the egg. However, variations in this wash time and lower temperatures enabled both *S.* Enteritidis and *S.* Typhimurium to penetrate the egg shell and contaminate the egg contents.

Egg washing protocols have been augmented by the addition of chemical compounds. Wang and Slavik [41] experimentally compared the effect three commercial egg washing compounds have on *Salmonella* penetration of the egg shell post washing. Two of the commercially available chemical compounds (quaternary ammonium compound (QAC, pH 7.5) and hypochlorite (NaOCl, 100 ppm, pH 7.5)) were shown to reduce *Salmonella* penetration of the egg shell; however, washing with sodium carbonate (Na_2_CO_3_, pH 12) was shown to facilitate bacterial penetration. Another addition to the egg washing protocol is the utilisation of slightly acidic electrolysed water, which was demonstrated by Cao *et al.* [43] to experimentally reduce and even eliminate *S.* Enteritidis on shell eggs and washing water preventing cross contamination. The difficulty with interpreting these results for real world application is that the effect on egg shell penetration was not explored.

Whole eggs and egg pulp pasteurisation through heat treating for short periods of time, is another method which has been demonstrated to reduce *Salmonella* contamination [40,44]. Barbour *et al.* [45] demonstrated that when whole eggs inoculated with *Salmonella* were treated by placing in a hot water bath at 57 °C for 25 min, followed by application of hot air at 55 °C for another 57 min a significant reduction in the *Salmonella* contamination was observed. When the initial inoculation was reduced to approximately 10^6^ CFU there were no viable *Salmonella* detected after this heat pasteurization [45]. Pasquali *et al.* [46] also demonstrated that hot air treatment only reduced *S.* Enteritidis contamination of eggshells by up to 1.9 log, with no significant changes to any of the quality traits of the egg. Pasteurisation may present an suitable method to reduce the risk of salmonellosis from eggs for high risk groups such as aged care facilities and hospitals; however, this is not likely to provide a solution for all due to consumers desire for raw whole foods and concern regarding consumption of pasteurised foods [47].

Irradiation of eggs has been presented as a potential method to prevent salmonellosis. The minimal dose required to inactivate *Salmonella* is 1.5 kGy which had been shown to cause changes in sensorial and functional properties of the egg. Sensorial changes include increased egg yolk odour and decreased clarity of the egg white, functional changes include decrease foam stability of the egg white [48]. Experimentally, egg whites irradiated at doses 2.5 and 5 kGy were shown to have increased foaming ability but decreased foam stability which obviously limits the functionality and desirability of the egg white [49]. Despite this, irradiation and pasteurisation may present an acceptable option for high risk populations, such as the elderly, immunocompromised, children and pregnant women. As such they may be a suitable control method against salmonellosis for hospitals and aged care facilities [48,50]. As such, regulatory guidelines enforcing the use of pasteurised egg products for vulnerable populations would be method to reduce the risk of salmonellosis.

Another approach was presented by Leleu *et al.* [51] who demonstrated that coating eggshell with chitosan (a linear polysaccharide derived from crustaceans) significantly reduced penetration by *S.* Enteritidis. Experimentally a 2% chitosan eggshell coating resulted in only 6.1% of eggs being penetrated compared to 24.5% of untreated eggs. However, chitosan coating did not reduce eggshell contamination, which does not prevent cross contamination during preparation to other food products.

## 8. Storage and Transport

A study by Radkowski [52] investigated the effect that storage at different temperatures had on 1,440 eggs with the outside of the shells artificially contaminated with 10 CFU of *S.* Enteritidis. The artificial contamination of shells occurred after 0, 10, or 20 days stored at room temperature and eggs were stored for 0, 7, 14 and 21 days at 2 °C, 20 °C, and 30 °C prior to measuring the remaining *S.* Enteritidis contamination. The results from this study showed that storage at lower temperatures actually increased *S.* Enteritidis survival on the outside of shell eggs. Alternatively Humphrey *et al.* [53] explored the effect that storage at room temperature has on the internal concentration of *S.* Enteritidis of contaminated eggs. During this study a total of 5262 hen eggs from 15 different *Salmonella* positive flocks were tested for *S.* Enteritidis at a varying number of days post laying during which they were stored at room temperature. In the first, second and third week post laying 5/1085 (0.5%), 7/1353 (0.5%) and 1/1221 (0.1%) of eggs were contaminated with *S.* Enteritidis and all contaminated eggs had <20 cells of *S.* Enteritidis. After 21 days though 12/1603 (0.8%) of eggs were *S.* Enteritidis positive, seven of these contained <20 cells but five had >100 cells with two eggs containing 1.5 × 10^4^ and 1.2 × 10^5^ cells of *S.* Enteritidis. Another study by Lublin *et al.* [54] demonstrated that after four weeks stored at 25 °C the concentration of *S.* Enteritidis in experimentally inoculated eggs began to increase. They also demonstrated the storing eggs at 6 °C prevented this increase in concentration observed at 25 °C, but did not prevent the survival of the initial concentration of *S.* Enteritidis. Egg storage at 10 °C and 20 °C was shown to control *S.* Enteritidis growth in experimentally inoculated eggs by Okamura *et al.* [55]. However, they also discovered that fluctuation in temperature promoted growth and that eggs stored at 22–30 °C or 27–35 °C for 5 days followed constant storage at 25 °C caused rapid increases in the number of eggs containing >10^6^ *S.* Enteritidis cells after only one and two weeks, respectively. This rapid increase due to fluctuation in temperature is important to consider what managing storage and transportation from the farm to the table.

## 9. Food Handling and Preparation

There are inconsistencies in the current literature regarding *Salmonella* control methods throughout the processing process and the evidence presented is conflicting. This places a lot of pressure on the control and management of *Salmonella* during food handling and preparation. The importance of management of this pathogen during food handing has been further increased by the growing desire of consumers for raw food products [16,17]. Humphrey *et al.* [56] demonstrated using a model kitchen and experimentally contaminated intact eggs that utensils used to mix eggs were sometimes *Salmonella* positive even after washing. When contaminated eggs were used in a batter mixture that was hand whisked with a fork or hand help mixer, *S.* Enteritidis was recovered from work surfaces over 40 cm away from the mixing bowl. Survival of the bacteria was seen in thin dry spots of egg or batter mixture 24 h after contamination. The importance of successfully cleaning kitchen to remove *Salmonella* contamination was also explored by Barker *et al.* [57]. In this study using detergent based cleaning without a rinse step was insufficient in achieving a hygienic surface within a model kitchen. Although the detergent based cleaning method was improved by addition of a rinse step, the use of hypochlorite at 5000 ppm was a significantly superior to the detergent based cleaning. This is an important message as sufficient cleaning of kitchen surfaces and utensils is crucial to prevent the cross contamination of *Salmonella* to other food products.

The importance of appropriate food handling is demonstrated by the example of a series of related salmonellosis outbreaks in Tasmania, Australia which occurred between June and December 2005. During this period there were five outbreaks and a total of 125 laboratory-confirmed cases of *S.* Typhimurium phage 135 reported to the Tasmania Department of Health. Of these cases 91% were linked to food businesses which had their eggs supplied from the same farm. Each business was found to have inadequate food handling and storage procedures which lead to the cross contamination of *Salmonella* [58]. Crespo *et al.* [59] investigated foodborne disease outbreaks in Spain associated with the consumption of eggs and egg products from 2000–2002. In total there were 895 outbreaks identified and of these 85% were attributed to *Salmonella* (of these 58% were confirmed as *S.* Enteritidis). Investigations into each of these outbreaks identified that the most important control measures was health education, followed by inspection of premises and monitoring of food handlers. The findings from these outbreaks demonstrated the responsibility of food handlers to ensure the hygiene of the finished food products and to never regard eggs as ‘sterile’. Many food handlers underestimate the risk of *Salmonella* from raw eggs [60]. A recent study interviewing head chefs and catering managers of restaurant in Owerri, Nigeria found that although all participants stated they washed their hands after handling raw meat, chicken or fish, 6% stated they did not wash their hands after cracking raw eggs [61].

The difficulty of ensuring food handlers have the appropriate information regarding food safety education is that the message is not always straight forward. For example Radford and Board [62] explored the role of homemade mayonnaise and *S.* Enteritidis survival. It was found that mayonnaise made with vinegar to a pH of 4.1 or less controlled *S.* Enteritidis. The addition of garlic and mustard to mayonnaise was also protective against *S.* Enteritidis; however, the addition of salt or any vegetable materials promoted the survival of *S.* Enteritidis. The types of oil and vinegar used affected the survival of *S.* Enteritidis and storage of the mayonnaise at refrigeration temperatures actually protected *Salmonella* from acidulants. It was recommended that the mayonnaise was stored at 18–22 °C for 24 h prior to refrigeration.

## 10. Conclusions

*Salmonella* contamination of eggs is a complex issue affected by variables at each stage of the food production process. Currently the literature regarding the benefits of free range, barn and caged production processes with respect to *Salmonella* contamination is conflicting. However, the current literature does indicate that it is not yet achievable to produce eggs guaranteed to be *Salmonella*-free. This reinforces the importance of post collection control measures for *Salmonella*. This includes post collection disinfection methods such as washing, pasteurisation and irradiation. Although the second two methods will not be desirable for all consumers, they provide a niche solution for high risk patients. There is also the need for further research to optimise storage, temperature and food handling protocols as currently the information is highly complex and variable. Given the current shift in consumers’ preference and increasing desire for raw food products, there is a need for more informed guidelines regarding the preparation of foods containing raw eggs. Further research is required to explore different protocols to ensure control of *Salmonella* through temperature and pH of food products. There is also a need to re-educate food handlers and consumers of the risk from raw eggs and cross contamination of food products and reduce the public health risk.
